# Bis[4-(2-benzoyl-1-oxidoethen­yl)-3-hy­droxy­phenyl benzoato]diethano­l­cobalt(II)

**DOI:** 10.1107/S1600536810029776

**Published:** 2010-07-31

**Authors:** Xue-Lian Bai, Tian-Li Yue, Ya-Hong Yuan, Hua-Wei Zhang

**Affiliations:** a College of Food Science and Engineering, Northwest A&F University, Yangling 712100, People’s Republic of China; b School of Pharmaceutical Sciences, Zhejiang University of Technology, Hangzhou 310014, People’s Republic of China

## Abstract

In the title complex, [Co(C_22_H_15_O_5_)_2_(C_2_H_5_OH)_2_], the Co^II^ atom (site symmetry 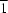) is coordinated by two *O*,*O*′-bidentate 4-(2-benzoyl-1-oxidoethen­yl)-3-hy­droxy­phenyl benzoate anions and two ethanol O atoms, resulting in a slightly distorted CoO_6_ octa­hedral coordination. An intra­molecular O—H⋯O hydrogen bond in the ligand generates an *S*(6) ring. The dihedral angle between the aromatic rings joined to the acetyl­acetonate unit is 6.4 (2)°. The ethanol mol­ecule is disordered over two orientations in a 0.65 (3):0.35 (3) ratio. In the crystal, mol­ecules are linked by O—H⋯O bonds.

## Related literature

For background to related cobalt complexes, see: Shi *et al.* (2008 ▶). For reference structural data, see: Allen *et al.* (1987 ▶).
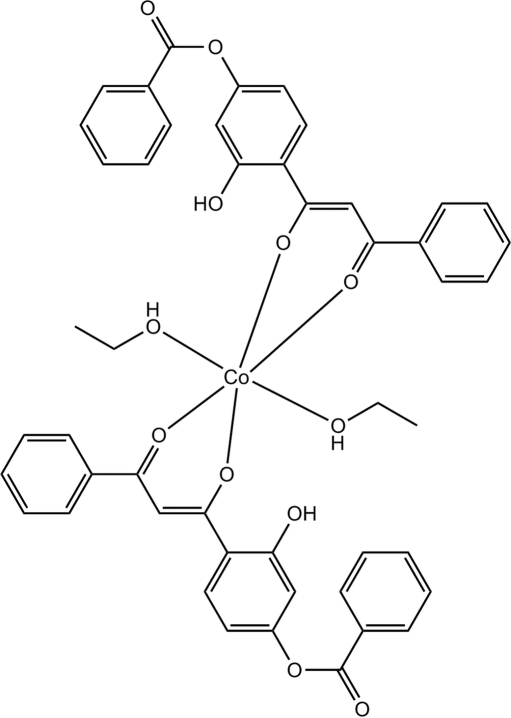


## Experimental

### 

#### Crystal data

[Co(C_22_H_15_O_5_)_2_(C_2_H_6_O)_2_]
*M* *_r_* = 869.75
Triclinic, 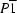

*a* = 7.2068 (9) Å
*b* = 9.4298 (12) Å
*c* = 16.5511 (19) Å
α = 106.358 (1)°
β = 95.431 (2)°
γ = 90.920 (1)°
*V* = 1073.3 (2) Å^3^
*Z* = 1
Mo *K*α radiation
μ = 0.46 mm^−1^
*T* = 298 K
0.38 × 0.15 × 0.10 mm

#### Data collection

Enraf–Nonius CAD-4 diffractometer
Absorption correction: ψ scan (North *et al.*, 1968 ▶) *T*_min_ = 0.844, *T*_max_ = 0.955
5612 measured reflections
3716 independent reflections
1953 reflections with *I* > 2σ(*I*)
*R*_int_ = 0.039
3 standard reflections every 200 reflections  intensity decay: 1%

#### Refinement

*R*[*F* ^2^ > 2σ(*F* ^2^)] = 0.065
*wR*(*F* ^2^) = 0.161
*S* = 0.95
3716 reflections
296 parameters
H-atom parameters constrained
Δρ_max_ = 0.33 e Å^−3^
Δρ_min_ = −0.39 e Å^−3^

### 

Data collection: *CAD-4 Software* (Enraf–Nonius, 1989 ▶); cell refinement: *CAD-4 Software*; data reduction: *XCAD4* (Harms & Wocadlo, 1995 ▶); program(s) used to solve structure: *SHELXS97* (Sheldrick, 2008 ▶); program(s) used to refine structure: *SHELXL97* (Sheldrick, 2008 ▶); molecular graphics: *SHELXTL* (Sheldrick, 2008 ▶); software used to prepare material for publication: *SHELXTL*.

## Supplementary Material

Crystal structure: contains datablocks global, I. DOI: 10.1107/S1600536810029776/hb5579sup1.cif

Structure factors: contains datablocks I. DOI: 10.1107/S1600536810029776/hb5579Isup2.hkl

Additional supplementary materials:  crystallographic information; 3D view; checkCIF report

## Figures and Tables

**Table 1:** Selected bond lengths (Å)

Co1—O1	2.015 (2)
Co1—O2	2.033 (3)
Co1—O6	2.184 (4)

**Table 2:** Hydrogen-bond geometry (Å, °)

*D*—H⋯*A*	*D*—H	H⋯*A*	*D*⋯*A*	*D*—H⋯*A*
O3—H3⋯O2	0.82	1.76	2.489 (4)	147
O6—H6⋯O3^i^	0.85	2.02	2.855 (4)	168

Symmetry code: (i) 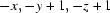.

**Fig. 1.**
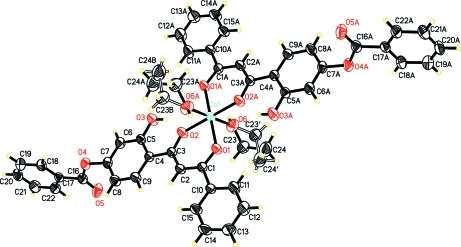
The molecular structure of (I) showing 30% probability displacement ellipsoids. Atoms with suffix A are generated by (1–x, 1–y, 1–z).

## supplementary crystallographic information

### Comment

There has been much research interest in cobalt complexes due to their molecular architectures and biological activities (Shi *et al.*, 2008). In this work, we report here the crystal structure of the title compound, (I). In (I), all bond lengths are within normal ranges (Allen *et al.*, 1987) (Fig. 1). The Co^II^ atom is six-coordinated by four O atoms from the 3-hydroxy-4-(1-hydroxy-3-oxo-3-phenylprop-1-enyl)phenyl benzoate and two O atoms from the ethanol molecules, forming a slightly distorted octahedral coordination. There is an intermolecuar and an intramolecular O—H···O hydrogen bonds in the title complex.

### Experimental

The title compound was prepared by stirring a mixture of 3-hydroxy-4-(1-hydroxy-3-oxo-3-phenylprop-1-enyl)phenyl benzoate (720 mg, 2 mmol) and CoCl_2_.6H_2_O (1 mmol, 238 mg) in ethanol (10 ml) for 3 h. After keeping the filtrate in air for 8 d, red block-shaped crystals of (I) were formed.

### Refinement

The N-bound H atom was located in a difference map and its position was freely refined. The other H atoms were positioned geometrically (C—H = 0.93–0.97 Å, O—H = 0.82 Å, S—H = 1.20 Å) and refined as riding, with *U*_iso_(H) = 1.2*U*_eq_(carrier) or 1.5*U*_eq_(methyl C).

### Crystal data

**Table:**

[Co(C_22_H_15_O_5_)_2_(C_2_H_6_O)_2_]	*Z* = 1
*M**_r_* = 869.75	*F*(000) = 453
Triclinic, *P*1	*D*_x_ = 1.346 Mg m^−^^3^
Hall symbol: -P 1	Mo *K*α radiation, λ = 0.71073 Å
*a* = 7.2068 (9) Å	Cell parameters from 25 reflections
*b* = 9.4298 (12) Å	θ = 9–12°
*c* = 16.5511 (19) Å	µ = 0.46 mm^−^^1^
α = 106.358 (1)°	*T* = 298 K
β = 95.431 (2)°	Block, red
γ = 90.920 (1)°	0.38 × 0.15 × 0.10 mm
*V* = 1073.3 (2) Å^3^	

### Data collection

**Table:**

Enraf–Nonius CAD-4 diffractometer	1953 reflections with *I* > 2σ(*I*)
Radiation source: fine-focus sealed tube	*R*_int_ = 0.039
graphite	θ_max_ = 25.0°, θ_min_ = 2.3°
ω/2θ scan	*h* = −8→7
Absorption correction: ψ scan (North *et al.*, 1968)	*k* = −11→11
*T*_min_ = 0.844, *T*_max_ = 0.955	*l* = −19→19
5612 measured reflections	3 standard reflections every 200 reflections
3716 independent reflections	intensity decay: 1%

### Refinement

**Table:**

Refinement on *F*^2^	Primary atom site location: structure-invariant direct methods
Least-squares matrix: full	Secondary atom site location: difference Fourier map
*R*[*F*^2^ > 2σ(*F*^2^)] = 0.065	Hydrogen site location: inferred from neighbouring sites
*wR*(*F*^2^) = 0.161	H-atom parameters constrained
*S* = 0.95	*w* = 1/[σ^2^(*F*_o_^2^) + (0.0689*P*)^2^] where *P* = (*F*_o_^2^ + 2*F*_c_^2^)/3
3716 reflections	(Δ/σ)_max_ = 0.001
296 parameters	Δρ_max_ = 0.33 e Å^−^^3^
0 restraints	Δρ_min_ = −0.39 e Å^−^^3^

### Special details

**Table:**

Geometry. All e.s.d.'s (except the e.s.d. in the dihedral angle between two l.s. planes) are estimated using the full covariance matrix. The cell e.s.d.'s are taken into account individually in the estimation of e.s.d.'s in distances, angles and torsion angles; correlations between e.s.d.'s in cell parameters are only used when they are defined by crystal symmetry. An approximate (isotropic) treatment of cell e.s.d.'s is used for estimating e.s.d.'s involving l.s. planes.
Refinement. Refinement of *F*^2^ against ALL reflections. The weighted *R*-factor *wR* and goodness of fit *S* are based on *F*^2^, conventional *R*-factors *R* are based on *F*, with *F* set to zero for negative *F*^2^. The threshold expression of *F*^2^ > σ(*F*^2^) is used only for calculating *R*-factors(gt) *etc*. and is not relevant to the choice of reflections for refinement. *R*-factors based on *F*^2^ are statistically about twice as large as those based on *F*, and *R*- factors based on ALL data will be even larger.

### Fractional atomic coordinates and isotropic or equivalent isotropic displacement parameters (Å^2^)

**Table:**

	*x*	*y*	*z*	*U*_iso_*/*U*_eq_	Occ. (<1)
Co1	0.5000	0.5000	0.5000	0.0624 (4)	
O1	0.6698 (4)	0.3797 (3)	0.55579 (17)	0.0628 (9)	
O2	0.3392 (4)	0.5269 (4)	0.59728 (17)	0.0651 (9)	
O3	0.0547 (4)	0.6632 (4)	0.63944 (17)	0.0761 (11)	
H3	0.1307	0.6221	0.6077	0.114*	
O4	−0.0989 (4)	0.7618 (4)	0.92129 (19)	0.0698 (9)	
O5	−0.2457 (5)	0.5537 (4)	0.9277 (2)	0.0889 (12)	
O6	0.3197 (5)	0.3103 (4)	0.4264 (2)	0.0835 (11)	
H6	0.2093	0.3309	0.4121	0.100*	
C1	0.6744 (6)	0.3714 (5)	0.6316 (3)	0.0496 (11)	
C2	0.5426 (6)	0.4316 (5)	0.6878 (2)	0.0553 (12)	
H2	0.5644	0.4225	0.7424	0.066*	
C3	0.3823 (6)	0.5036 (5)	0.6703 (2)	0.0487 (11)	
C4	0.2513 (5)	0.5608 (5)	0.7353 (2)	0.0481 (11)	
C5	0.0949 (6)	0.6410 (5)	0.7169 (2)	0.0507 (11)	
C6	−0.0226 (6)	0.7014 (5)	0.7786 (3)	0.0587 (12)	
H6A	−0.1229	0.7551	0.7661	0.070*	
C7	0.0089 (6)	0.6822 (5)	0.8575 (3)	0.0582 (12)	
C8	0.1538 (6)	0.5989 (5)	0.8769 (3)	0.0658 (14)	
H8	0.1708	0.5818	0.9297	0.079*	
C9	0.2720 (6)	0.5420 (5)	0.8164 (3)	0.0597 (13)	
H9	0.3710	0.4882	0.8302	0.072*	
C10	0.8321 (6)	0.2857 (5)	0.6592 (2)	0.0510 (12)	
C11	0.9469 (7)	0.2133 (6)	0.6006 (3)	0.0824 (18)	
H11	0.9284	0.2200	0.5454	0.099*	
C12	1.0905 (8)	0.1300 (7)	0.6226 (3)	0.100 (2)	
H12	1.1665	0.0814	0.5821	0.120*	
C13	1.1205 (8)	0.1191 (6)	0.7038 (3)	0.0886 (18)	
H13	1.2125	0.0597	0.7181	0.106*	
C14	1.0137 (8)	0.1964 (6)	0.7625 (3)	0.0873 (19)	
H14	1.0363	0.1935	0.8182	0.105*	
C15	0.8721 (7)	0.2792 (6)	0.7409 (3)	0.0714 (15)	
H15	0.8016	0.3322	0.7827	0.086*	
C16	−0.2220 (7)	0.6860 (6)	0.9529 (3)	0.0588 (12)	
C17	−0.3227 (6)	0.7882 (5)	1.0192 (2)	0.0515 (12)	
C18	−0.2748 (7)	0.9379 (6)	1.0524 (3)	0.0638 (13)	
H18	−0.1730	0.9787	1.0348	0.077*	
C19	−0.3782 (7)	1.0260 (6)	1.1114 (3)	0.0751 (15)	
H19	−0.3452	1.1261	1.1341	0.090*	
C20	−0.5292 (8)	0.9668 (7)	1.1368 (3)	0.0784 (17)	
H20	−0.5986	1.0270	1.1764	0.094*	
C21	−0.5791 (8)	0.8193 (7)	1.1042 (3)	0.0843 (17)	
H21	−0.6831	0.7798	1.1208	0.101*	
C22	−0.4739 (7)	0.7297 (6)	1.0465 (3)	0.0697 (14)	
H22	−0.5051	0.6290	1.0258	0.084*	
C23	0.3217 (17)	0.1644 (13)	0.4320 (14)	0.087 (5)	0.65 (3)
H23A	0.2041	0.1119	0.4068	0.104*	0.65 (3)
H23B	0.3398	0.1644	0.4909	0.104*	0.65 (3)
C24	0.479 (6)	0.090 (5)	0.3856 (19)	0.116 (8)	0.65 (3)
H24A	0.4836	0.1185	0.3344	0.174*	0.65 (3)
H24B	0.4601	−0.0151	0.3719	0.174*	0.65 (3)
H24C	0.5951	0.1202	0.4207	0.174*	0.65 (3)
C23'	0.348 (3)	0.165 (2)	0.375 (3)	0.087 (8)	0.35 (3)
H23C	0.4117	0.1730	0.3273	0.104*	0.35 (3)
H23D	0.2272	0.1159	0.3526	0.104*	0.35 (3)
C24'	0.458 (11)	0.067 (9)	0.418 (3)	0.116 (14)	0.35 (3)
H24D	0.5487	0.1267	0.4610	0.174*	0.35 (3)
H24E	0.5215	−0.0033	0.3770	0.174*	0.35 (3)
H24F	0.3755	0.0156	0.4433	0.174*	0.35 (3)

### Atomic displacement parameters (Å^2^)

**Table:**

	*U*^11^	*U*^22^	*U*^33^	*U*^12^	*U*^13^	*U*^23^
Co1	0.0504 (6)	0.0955 (8)	0.0497 (5)	0.0298 (5)	0.0152 (4)	0.0298 (5)
O1	0.053 (2)	0.094 (2)	0.0492 (17)	0.0336 (18)	0.0160 (15)	0.0283 (17)
O2	0.053 (2)	0.102 (3)	0.0502 (17)	0.0321 (18)	0.0154 (15)	0.0323 (17)
O3	0.056 (2)	0.123 (3)	0.0579 (19)	0.044 (2)	0.0174 (16)	0.0331 (19)
O4	0.064 (2)	0.072 (2)	0.077 (2)	0.0116 (18)	0.0381 (18)	0.0150 (18)
O5	0.093 (3)	0.066 (3)	0.104 (3)	0.003 (2)	0.051 (2)	0.005 (2)
O6	0.058 (2)	0.090 (3)	0.100 (3)	0.027 (2)	−0.002 (2)	0.024 (2)
C1	0.040 (3)	0.061 (3)	0.049 (3)	0.014 (2)	0.005 (2)	0.017 (2)
C2	0.045 (3)	0.080 (3)	0.048 (2)	0.022 (3)	0.013 (2)	0.027 (2)
C3	0.039 (3)	0.061 (3)	0.048 (2)	0.011 (2)	0.008 (2)	0.017 (2)
C4	0.034 (2)	0.062 (3)	0.051 (2)	0.012 (2)	0.010 (2)	0.016 (2)
C5	0.041 (3)	0.067 (3)	0.045 (2)	0.009 (2)	0.010 (2)	0.015 (2)
C6	0.042 (3)	0.074 (3)	0.062 (3)	0.018 (2)	0.018 (2)	0.018 (2)
C7	0.048 (3)	0.065 (3)	0.062 (3)	0.010 (3)	0.023 (2)	0.012 (2)
C8	0.058 (3)	0.088 (4)	0.058 (3)	0.014 (3)	0.020 (3)	0.027 (3)
C9	0.049 (3)	0.083 (4)	0.055 (3)	0.024 (3)	0.016 (2)	0.029 (2)
C10	0.043 (3)	0.067 (3)	0.049 (3)	0.014 (2)	0.010 (2)	0.023 (2)
C11	0.073 (4)	0.123 (5)	0.057 (3)	0.053 (4)	0.019 (3)	0.028 (3)
C12	0.082 (4)	0.142 (6)	0.083 (4)	0.074 (4)	0.025 (3)	0.034 (4)
C13	0.078 (4)	0.115 (5)	0.082 (4)	0.045 (4)	0.004 (3)	0.042 (3)
C14	0.085 (4)	0.129 (5)	0.066 (3)	0.052 (4)	0.014 (3)	0.052 (3)
C15	0.065 (3)	0.104 (4)	0.058 (3)	0.035 (3)	0.017 (3)	0.038 (3)
C16	0.051 (3)	0.071 (4)	0.058 (3)	0.007 (3)	0.018 (2)	0.018 (3)
C17	0.048 (3)	0.064 (3)	0.044 (2)	0.010 (3)	0.011 (2)	0.017 (2)
C18	0.055 (3)	0.076 (4)	0.059 (3)	0.004 (3)	0.018 (3)	0.013 (3)
C19	0.074 (4)	0.075 (4)	0.068 (3)	0.006 (3)	0.013 (3)	0.006 (3)
C20	0.084 (4)	0.096 (5)	0.057 (3)	0.026 (4)	0.033 (3)	0.016 (3)
C21	0.079 (4)	0.100 (5)	0.086 (4)	0.009 (4)	0.047 (3)	0.032 (4)
C22	0.069 (3)	0.071 (4)	0.074 (3)	0.006 (3)	0.032 (3)	0.020 (3)
C23	0.072 (7)	0.096 (9)	0.094 (10)	0.027 (6)	0.011 (7)	0.030 (7)
C24	0.110 (14)	0.114 (16)	0.12 (2)	0.037 (12)	0.017 (16)	0.017 (14)
C23'	0.072 (13)	0.097 (16)	0.09 (2)	0.027 (11)	0.011 (12)	0.030 (13)
C24'	0.11 (3)	0.11 (3)	0.12 (4)	0.04 (2)	0.02 (3)	0.02 (3)

### Geometric parameters (Å, °)

**Table:**

Co1—O1^i^	2.015 (2)	C11—H11	0.9300
Co1—O1	2.015 (2)	C12—C13	1.373 (6)
Co1—O2^i^	2.033 (3)	C12—H12	0.9300
Co1—O2	2.033 (3)	C13—C14	1.352 (6)
Co1—O6^i^	2.184 (4)	C13—H13	0.9300
Co1—O6	2.184 (4)	C14—C15	1.376 (6)
O1—C1	1.276 (4)	C14—H14	0.9300
O2—C3	1.299 (4)	C15—H15	0.9300
O3—C5	1.361 (4)	C16—C17	1.495 (6)
O3—H3	0.8200	C17—C22	1.382 (6)
O4—C16	1.360 (5)	C17—C18	1.388 (6)
O4—C7	1.417 (5)	C18—C19	1.379 (6)
O5—C16	1.202 (5)	C18—H18	0.9300
O6—C23	1.405 (13)	C19—C20	1.368 (7)
O6—C23'	1.43 (2)	C19—H19	0.9300
O6—H6	0.8500	C20—C21	1.371 (7)
C1—C2	1.406 (5)	C20—H20	0.9300
C1—C10	1.514 (5)	C21—C22	1.379 (6)
C2—C3	1.397 (5)	C21—H21	0.9300
C2—H2	0.9300	C22—H22	0.9300
C3—C4	1.490 (5)	C23—C24	1.50 (5)
C4—C9	1.397 (5)	C23—H23A	0.9700
C4—C5	1.425 (5)	C23—H23B	0.9700
C5—C6	1.392 (5)	C24—H24A	0.9600
C6—C7	1.366 (6)	C24—H24B	0.9600
C6—H6A	0.9300	C24—H24C	0.9600
C7—C8	1.384 (6)	C23'—C24'	1.51 (8)
C8—C9	1.375 (5)	C23'—H23C	0.9700
C8—H8	0.9300	C23'—H23D	0.9700
C9—H9	0.9300	C24'—H24D	0.9600
C10—C11	1.374 (5)	C24'—H24E	0.9600
C10—C15	1.375 (5)	C24'—H24F	0.9600
C11—C12	1.391 (6)		
			
O1^i^—Co1—O1	180.00 (13)	C13—C12—C11	120.4 (5)
O1^i^—Co1—O2^i^	88.35 (11)	C13—C12—H12	119.8
O1—Co1—O2^i^	91.65 (11)	C11—C12—H12	119.8
O1^i^—Co1—O2	91.65 (11)	C14—C13—C12	118.6 (4)
O1—Co1—O2	88.35 (11)	C14—C13—H13	120.7
O2^i^—Co1—O2	180.000 (1)	C12—C13—H13	120.7
O1^i^—Co1—O6^i^	94.47 (13)	C13—C14—C15	120.9 (4)
O1—Co1—O6^i^	85.53 (13)	C13—C14—H14	119.5
O2^i^—Co1—O6^i^	89.66 (13)	C15—C14—H14	119.5
O2—Co1—O6^i^	90.34 (13)	C10—C15—C14	121.7 (4)
O1^i^—Co1—O6	85.53 (13)	C10—C15—H15	119.2
O1—Co1—O6	94.47 (13)	C14—C15—H15	119.2
O2^i^—Co1—O6	90.34 (13)	O5—C16—O4	122.5 (4)
O2—Co1—O6	89.66 (13)	O5—C16—C17	126.1 (5)
O6^i^—Co1—O6	180.000 (1)	O4—C16—C17	111.4 (5)
C1—O1—Co1	127.4 (2)	C22—C17—C18	119.1 (4)
C3—O2—Co1	127.9 (3)	C22—C17—C16	117.6 (5)
C5—O3—H3	109.5	C18—C17—C16	123.3 (4)
C16—O4—C7	119.1 (4)	C19—C18—C17	119.9 (5)
C23—O6—C23'	40.5 (9)	C19—C18—H18	120.0
C23—O6—Co1	128.0 (6)	C17—C18—H18	120.0
C23'—O6—Co1	135.4 (9)	C20—C19—C18	120.2 (5)
C23—O6—H6	110.0	C20—C19—H19	119.9
C23'—O6—H6	106.9	C18—C19—H19	119.9
Co1—O6—H6	115.0	C19—C20—C21	120.5 (5)
O1—C1—C2	124.7 (4)	C19—C20—H20	119.7
O1—C1—C10	115.6 (3)	C21—C20—H20	119.7
C2—C1—C10	119.7 (3)	C20—C21—C22	119.6 (5)
C3—C2—C1	126.8 (4)	C20—C21—H21	120.2
C3—C2—H2	116.6	C22—C21—H21	120.2
C1—C2—H2	116.6	C21—C22—C17	120.6 (5)
O2—C3—C2	122.5 (4)	C21—C22—H22	119.7
O2—C3—C4	115.8 (3)	C17—C22—H22	119.7
C2—C3—C4	121.7 (3)	O6—C23—C24	108 (2)
C9—C4—C5	115.9 (4)	O6—C23—H23A	110.2
C9—C4—C3	123.8 (4)	C24—C23—H23A	110.2
C5—C4—C3	120.3 (3)	O6—C23—H23B	110.2
O3—C5—C6	117.3 (4)	C24—C23—H23B	110.2
O3—C5—C4	122.1 (4)	H23A—C23—H23B	108.5
C6—C5—C4	120.6 (4)	C23—C24—H24A	109.5
C7—C6—C5	120.3 (4)	C23—C24—H24B	109.5
C7—C6—H6A	119.8	H24A—C24—H24B	109.5
C5—C6—H6A	119.8	C23—C24—H24C	109.5
C6—C7—C8	121.0 (4)	H24A—C24—H24C	109.5
C6—C7—O4	118.1 (4)	H24B—C24—H24C	109.5
C8—C7—O4	120.6 (4)	O6—C23'—C24'	116 (4)
C9—C8—C7	118.5 (4)	O6—C23'—H23C	108.2
C9—C8—H8	120.7	C24'—C23'—H23C	108.2
C7—C8—H8	120.7	O6—C23'—H23D	108.2
C8—C9—C4	123.5 (4)	C24'—C23'—H23D	108.2
C8—C9—H9	118.2	H23C—C23'—H23D	107.4
C4—C9—H9	118.2	C23'—C24'—H24D	109.5
C11—C10—C15	117.2 (4)	C23'—C24'—H24E	109.5
C11—C10—C1	119.0 (4)	H24D—C24'—H24E	109.5
C15—C10—C1	123.8 (4)	C23'—C24'—H24F	109.5
C10—C11—C12	121.0 (4)	H24D—C24'—H24F	109.5
C10—C11—H11	119.5	H24E—C24'—H24F	109.5
C12—C11—H11	119.5		
			
O1^i^—Co1—O1—C1	−81 (100)	C5—C6—C7—O4	171.8 (4)
O2^i^—Co1—O1—C1	165.4 (4)	C16—O4—C7—C6	113.6 (5)
O2—Co1—O1—C1	−14.6 (4)	C16—O4—C7—C8	−72.5 (6)
O6^i^—Co1—O1—C1	75.9 (4)	C6—C7—C8—C9	3.4 (8)
O6—Co1—O1—C1	−104.1 (4)	O4—C7—C8—C9	−170.3 (4)
O1^i^—Co1—O2—C3	−164.3 (4)	C7—C8—C9—C4	−1.6 (8)
O1—Co1—O2—C3	15.7 (4)	C5—C4—C9—C8	−1.5 (7)
O2^i^—Co1—O2—C3	108 (100)	C3—C4—C9—C8	178.0 (4)
O6^i^—Co1—O2—C3	−69.8 (4)	O1—C1—C10—C11	−5.6 (7)
O6—Co1—O2—C3	110.2 (4)	C2—C1—C10—C11	172.5 (5)
O1^i^—Co1—O6—C23	−172.1 (11)	O1—C1—C10—C15	172.3 (5)
O1—Co1—O6—C23	7.9 (11)	C2—C1—C10—C15	−9.5 (7)
O2^i^—Co1—O6—C23	99.5 (11)	C15—C10—C11—C12	3.6 (8)
O2—Co1—O6—C23	−80.5 (11)	C1—C10—C11—C12	−178.3 (5)
O6^i^—Co1—O6—C23	−19 (100)	C10—C11—C12—C13	−0.2 (9)
O1^i^—Co1—O6—C23'	133 (2)	C11—C12—C13—C14	−3.1 (10)
O1—Co1—O6—C23'	−47 (2)	C12—C13—C14—C15	2.9 (10)
O2^i^—Co1—O6—C23'	45 (2)	C11—C10—C15—C14	−3.9 (8)
O2—Co1—O6—C23'	−135 (2)	C1—C10—C15—C14	178.1 (5)
O6^i^—Co1—O6—C23'	−74 (100)	C13—C14—C15—C10	0.7 (9)
Co1—O1—C1—C2	8.8 (7)	C7—O4—C16—O5	−1.4 (7)
Co1—O1—C1—C10	−173.2 (3)	C7—O4—C16—C17	−179.9 (3)
O1—C1—C2—C3	3.0 (8)	O5—C16—C17—C22	−9.3 (7)
C10—C1—C2—C3	−175.0 (4)	O4—C16—C17—C22	169.2 (4)
Co1—O2—C3—C2	−10.7 (6)	O5—C16—C17—C18	172.5 (5)
Co1—O2—C3—C4	168.4 (3)	O4—C16—C17—C18	−9.0 (6)
C1—C2—C3—O2	−1.9 (8)	C22—C17—C18—C19	−0.3 (7)
C1—C2—C3—C4	179.0 (4)	C16—C17—C18—C19	177.9 (4)
O2—C3—C4—C9	178.0 (4)	C17—C18—C19—C20	−0.7 (7)
C2—C3—C4—C9	−3.0 (7)	C18—C19—C20—C21	0.3 (8)
O2—C3—C4—C5	−2.6 (6)	C19—C20—C21—C22	1.2 (8)
C2—C3—C4—C5	176.4 (4)	C20—C21—C22—C17	−2.2 (8)
C9—C4—C5—O3	−177.8 (4)	C18—C17—C22—C21	1.7 (7)
C3—C4—C5—O3	2.7 (7)	C16—C17—C22—C21	−176.6 (4)
C9—C4—C5—C6	2.8 (7)	C23'—O6—C23—C24	40 (2)
C3—C4—C5—C6	−176.6 (4)	Co1—O6—C23—C24	−79 (2)
O3—C5—C6—C7	179.4 (4)	C23—O6—C23'—C24'	−40 (4)
C4—C5—C6—C7	−1.2 (7)	Co1—O6—C23'—C24'	59 (5)
C5—C6—C7—C8	−2.0 (8)		

Symmetry codes: (i) −*x*+1, −*y*+1, −*z*+1.

### Hydrogen-bond geometry (Å, °)

**Table:**

*D*—H···*A*	*D*—H	H···*A*	*D*···*A*	*D*—H···*A*
O3—H3···O2	0.82	1.76	2.489 (4)	147
O6—H6···O3^ii^	0.85	2.02	2.855 (4)	168

Symmetry codes: (ii) −*x*, −*y*+1, −*z*+1.
